# Preoperative Evaluation with fMRI of Patients with Intracranial Gliomas

**DOI:** 10.1155/2012/727810

**Published:** 2012-07-12

**Authors:** Ioannis Z. Kapsalakis, Eftychia Z. Kapsalaki, Efstathios D. Gotsis, Dimitrios Verganelakis, Panagiotis Toulas, Georgios Hadjigeorgiou, Indug Chung, Ioannis Fezoulidis, Alexandros Papadimitriou, Joe Sam Robinson, Gregory P. Lee, Kostas N. Fountas

**Affiliations:** ^1^Department of Neurology, School of Medicine, University Hospital of Larisa, University of Thessaly, 41110 Larisa, Greece; ^2^Department of Diagnostic Radiology, School of Medicine, University Hospital of Larisa, University of Thessaly, 41110 Larisa, Greece; ^3^Department of MR Imaging, Advanced Diagnostic and Research Institute “Euromedica-Encephalos”, 15233 Athens, Greece; ^4^Departments of Neurosurgery and Intraoperative Electrophysiology, Medical Center of Central Georgia, School of Medicine, Mercer University, Macon, GA 31201, USA; ^5^Department of Neurology, Medical College of Georgia, Augusta, GA 30912, USA; ^6^Department of Neurosurgery, School of Medicine, University Hospital of Larisa, University of Thessaly, 41110 Larisa, Greece; ^7^Institute of Biomedical Research and Technology (BIOMED), Center for Research and Technology-Thessaly (CERETETH), 38500 Larissa, Greece

## Abstract

*Introduction*. Aggressive surgical resection constitutes the optimal treatment for intracranial gliomas. However, the proximity of a tumor to eloquent areas requires exact knowledge of its anatomic relationships to functional cortex. The purpose of our study was to evaluate fMRI's accuracy by comparing it to intraoperative cortical stimulation (DCS) mapping. *Material and Methods*. Eighty-seven patients, with presumed glioma diagnosis, underwent preoperative fMRI and intraoperative DCS for cortical mapping during tumor resection. Findings of fMRI and DCS were considered concordant if the identified cortical centers were less than 5 mm apart. Pre and postoperative Karnofsky Performance Scale and Spitzer scores were recorded. A postoperative MRI was obtained for assessing the extent of resection. *Results*. The areas of interest were identified by fMRI and DCS in all participants. The concordance between fMRI and DCS was 91.9% regarding sensory-motor cortex, 100% for visual cortex, and 85.4% for language. Data analysis showed that patients with better functional condition demonstrated higher concordance rates, while there also was a weak association between tumor grade and concordance rate. The mean extent of tumor resection was 96.7%. *Conclusions*. Functional MRI is a highly accurate preoperative methodology for sensory-motor mapping. However, in language mapping, DCS remains necessary for accurate localization.

## 1. Introduction

Resection of brain tumors involving eloquent cortical areas has remained a challenging task [1–6]. Preservation of neuronal functions after surgery remains the goal for patients with primary and/or metastatic tumors involving the central, visual, Broca's, and/or Wernicke's areas. Intraoperative electric direct cortical stimulation (DCS) and mapping can accurately identify and define eloquent cortical areas, can examine their spatial relationships with the tumor, and can facilitate aggressive tumor resection [3, 4, 7–11]. However, DCS mapping requires either an awake craniotomy and a cooperative patient, at least for language area mapping, or a second operative procedure for extraoperative cortical stimulation and mapping via previously implanted subdural electrodes [4, 12–15]. In addition, DCS can identify cortical language-associated areas but cannot easily outline subcortical or intrasulcal speech areas [16, 17].

 Functional MRI (fMRI) is a noninvasive, imaging modality that has been used for mapping regions of the brain associated with motor, sensory, language, vision, and other cognitive tasks [18–25]. The change in signal detected by fMRI as neuronal activation is presumed to result from changes in regional, temporary concentrations of oxyhemoglobin caused by increased regional blood flow [26]. It has been extensively described that fMRI is based on a complex physiological phenomenon called Blood-Oxygenation-Level-Dependent (BOLD) effect [26]. The employment of fMRI in the presurgical planning of patients with brain tumors adjacent or in eloquent cortical areas has been increasing, in order to minimize the possibility of postoperative neurological deficit while may maximize the extent of tumor resection [6, 27–59]. An exponentially increasing number of clinical investigators have been applying various fMRI paradigms and protocols in patients with primary or metastatic tumors for identifying preoperatively their relationship with eloquent cortical areas [6, 27–59]. However, there is a significant variation in the reported fMRI accuracy rates, and frequently conflicting conclusions regarding the value of fMRI in the preoperative evaluation of patients with intracranial tumors [6, 27–59].

 In our current study, we present our results from employing both preoperative fMRI and intraoperative electric DCS on patients undergoing craniotomy for supratentorial glioma resection. The accuracy of the preoperative fMRI was compared to the intraoperative electrophysiologic findings, and the overall role of fMRI in intracranial glioma surgery was evaluated.

## 2. Material and Methods

 Our prospective clinical study covered a 10-year period (2002–2011). The study was approved by the Institutional Review Board of all the participating institutions. A signed written consent form was obtained from the participants or their legal representatives. The analysis of our data was performed according to the regulations of the current Health Insurance Portability and Accountability Act.

 The inclusion criteria in our study were (i) patient's age >18 years, (ii) presence of novel, supratentorial, presumably glial tumor (based on conventional MRI and proton MR spectroscopy whenever available), (iii) tumor location within or in the close proximity of eloquent cortical area, and (iv) patient's consent to participate in the study protocol. Patients with recurrent tumors, or previously irradiated tumors, were excluded from our study.

 A total of 892 patients with supratentorial tumors were evaluated during the study period in the participating institutions. However, only 87 patients (53 males and 34 females) met our inclusion criteria and participated in our study (Table 1). Their ages ranged between 33 and 76 years (mean age: 62.8). Detailed pre and postoperative neurological examinations, as well as neuropsychological (Mini Mental Status Examination), performance scores (Karnofsky Performance Scale, KPS, scores), and quality of daily life (Spitzer Quality of Life Index) evaluations were obtained in all cases. The postoperative KPS, and Spitzer indices were obtained one month after the patients' discharge.

**Figure 1:**
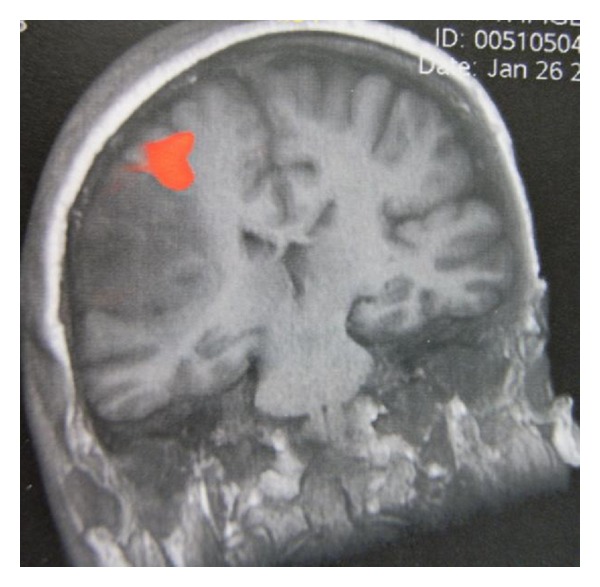
fMRI showing right hand activation antero-superiorly to the lesion.

 A conventional brain MRI study in a 1.5 T MRI scanner was obtained in all patients. An fMRI study was also obtained in all participants. The study was obtained within a month prior to their scheduled surgery. Foam cushions and straps were used for comfortably immobilizing the patient's head. BRAVO pulse sequence was utilized for obtaining the 3-dimensional anatomical images (248 images, flip angle = 15°, TE = 3.7 ms, TR = 9 ms, Th0/Sp = 1.4/−0.7 mm, FOV: 26 × 26). The following motor tasks were used: (i) a finger-thumb tapping test, (ii) periodic fist clenching/spreading test, (iii) periodic active movement of the foot, and (iv) periodic circular movements of the tongue (Figure 1). Tactile stimulation of the face, the hand, and/or the foot with a toothbrush was used for identifying the postcentral gyrus. The following verbal tasks were used for identifying the language associated cortical areas: (i) picture naming, (ii) word listening and parroting also, (iii) production of a noun from a verb or vice versa, (iv) finding a word of the opposite meaning in a given word, (v) reciting a well-known poem or a song, (iv) performance of simple mathematical calculations, (vii) countdown from 100 by subtracting 7. Each task performance test lasted 2.5 min including two periods of activation interspersed with three periods of rest of 30 sec duration each. In addition, a head CT scan was also obtained the day before the procedure for image fusion purposes and for ruling out the possibility of magnetic image distortion.

**Figure 2:**
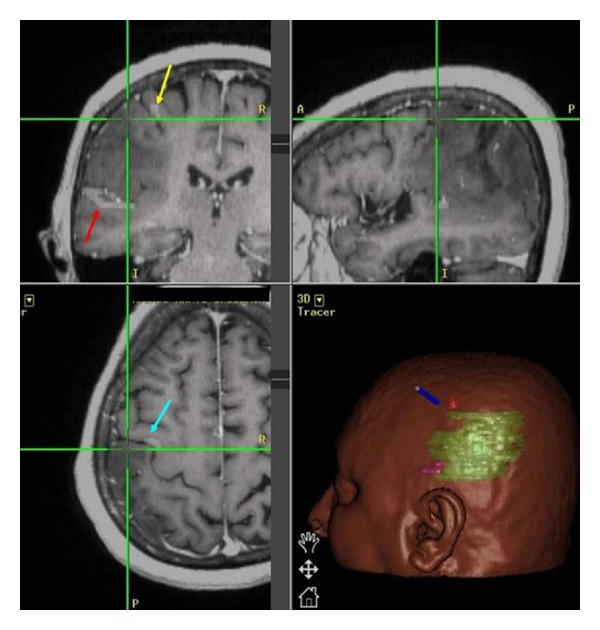
Neuronavigation workstation reproduced image showing superimposition of the fMRI data on the MR images. Red arrow shows the language area, yellow arrow shows right-hand motor area, and blue arrow shows (R hand) activation just anterior to the central sulcus.

 A StealthStation S7 neuro-navigational system (Medtronic, Minneapolis, MN, USA) was used in all our cases. The MRI, fMRI, and CT scans of each patient were uploaded, on the system's workstation, and image fusion was routinely performed (Figure 2). The accuracy of the system was also tested by identifying well-known anatomical landmarks, such as the osseous nasal bridge, the orbital rim, the medial and lateral canthi, and/or the acoustic meatus.

**Figure 3:**
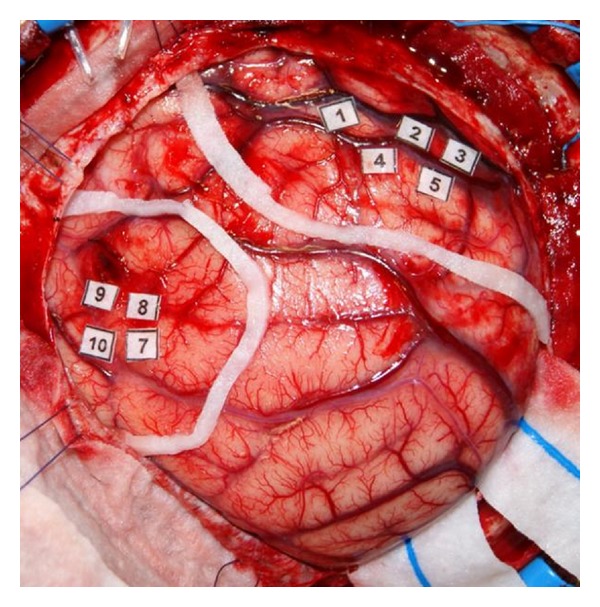
Intraoperative photo showing areas of language-associated activation (stickers 7–10) and right-hand motor activation (stickers 1–5). Note that eloquent areas with 1 cm safety margin have been outlined by a white cottonoid strap.

Intraoperative spontaneous electromyography (EMG), as well as motor, and somatosensory evoked potentials were employed in all our cases. In cases of language mapping awake craniotomy was performed in 31/48 cases (64.6%), while an extraoperative language mapping via previously implanted subdural grid and strip electrodes was performed in the remaining 17 cases (35.4%). Furthermore, DCS was performed by using an Ojemann bipolar stimulator with constant electric current for stimulation (frequency: 60 Hz, pulse width: 1 msec, train: 3 secs, amplitude: 4–12 mAmp) (Figure 3). Picture naming (flash card demonstration), rehearsing, word listening and parroting, opposite word finding, production of a noun from a verb or vice versa, counting, and countdown from 100 by subtracting 7 were utilized in our language mapping cases. In the case that after-discharges were observed, stimulation was temporarily suspended and the amplitude of the applied stimulus was promptly reduced. Pre and postcentral gyri were successfully identified in all cases, while Broca's and Wernicke's areas in 48/48 patients (Table 1).

 Special attention was paid during the surgical procedure for avoiding any losses of even minimal amounts of CSF, which could result in brain shift and localization inaccuracies. No osmotic agents or diuretics were used in any of our cases before defining the cortical eloquent areas and outlining the tumor. Moreover, the routine employment of somatosensory and motor evoked potential neuromonitoring confirmed the intraoperative accuracy of the navigator by identifying the central sulcus on each case.

**Figure 4:**
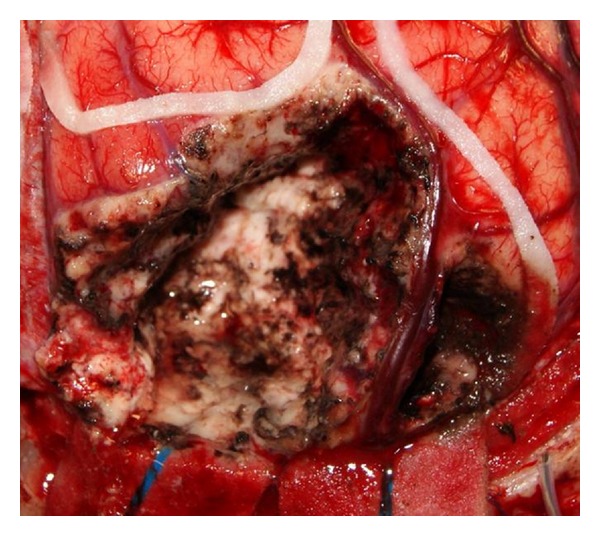
Intraoperative photo demonstrating the postresection cavity. The outlined eloquent areas have been preserved as well as the large cortical venous structures in the tumor bed.

 After careful dural opening and prompt identification of the tumor, its contour was outlined on the cortical surface by employing a postfencing technique for which tips of ventricular catheters were utilized for defining the tumor's perimetry. Subpial dissection, aspiration, and resection techniques were used for tumor removal (Figure 4). Furthermore, after completing the tumor resection, sampling biopsies were performed at positions 3, 6, 9, 12 o'clock of the resection cavity for ruling out any further tumor infiltration.

The findings of fMRI and intraoperative electrophysiologic monitoring regarding the localization of motor, sensory, visual, and language cortical areas were compared. If the fMRI and cortical stimulation areas were exactly the same or less than 5 mm apart, these studies were considered concordant. In those cases that the distance between the fMRI and the DCS defined eloquent areas was more than 5 mm, the employed studies were considered nonconcordant.

Statistical analysis of our data was performed by employing ANOVA, Fisher, and *χ* ^2^ statistical tests, while the level of significance was set at 5%. The histopathological reports were recorded in all our cases. An immediate postoperative MRI was obtained (within 72 hours from surgery) for assessing the extent of tumor resection, as well as for establishing a baseline for administrating any further adjuvant treatments. It has to be pointed out that in cases of enhancing lesions removal of the enhancing parts on postoperative MRI was considered as total removal, while in cases of non-enhancing lesions the extent of resection was based on the T2 weighted postoperative images.

## 3. Results

The anatomic location of the tumors in our current series was as follows: (i) 20 patients (23.0%) had left frontal tumors, (ii) 25 patients (28.7%) had left temporal tumors, (iii) 3 patients (3.4%) had right temporal tumors, (iv) 17 patients (19.5%) had left parietal tumors, (v) 20 patients (23.0%) had right parietal tumors, and (vi) 2 patients (2.3%) had left occipital tumors. Analytical data regarding the patients' demographic data, their tumor anatomic location, their pre and postoperative MMSE and Spitzer index scores are summarized in Table 1.

 The mean preoperative KPS score was 91.7 (range: 70–100), while the respective postoperative one was 94.9 (range: 70–100). The mean preoperative Spitzer index was 8.9 (range: 6–10), while the respective postoperative one was 9.1 (range: 6–10). There was worsening of the preoperative KPS score in 2/87 (2.3%) of our cases, while the respective percentage for the postoperative Spitzer indices was 1.1% (1/87 cases). Analysis of our current dataset confirmed that there was a statistically significant association between the patient's age and the tumor histological type (*F* value = 30.7, *P* value ≤ 0.0001). Thus, oligodendrogliomas and low grade astrocytomas occurred, as expected in younger patients, while anaplastic astrocytomas in more elderly patients and GBM in even older patients. Contrariwise, there was no statistically significant association between the anatomic location and the tumor histological type in our cohort (*χ* ^2^ = 8.445, *P* value = 0.5854). Moreover, there was a relationship between the patient's age and the occurrence of postoperative neurological deficits. Statistical analysis showed a trend for younger patients to develop less frequently postoperative neurological deficits (*F* value = 1.627, *P* value = 0.0611). A strong relationship was demonstrated between the tumor's anatomic location and the occurrence of postoperative neurological deficits. Patients with tumors located in the right temporal or the occipital lobes developed less frequently postoperative neurological deficits (*χ* ^2^ = 216.993, *P* value ≤ 0.0001).

In regard to the preoperative fMRI results, the pre and postcentral gyri were identified in all participants. Likewise, Broca's and Wernicke's areas were successfully identified in all our cases requiring language mapping. All tests were performed in the patient's native language (either English or Greek) without testing any additional languages. The visual cortex was successfully identified and was outlined in the obtained fMRI studies in both occipital cases in our series. Intraoperative DCS along with visual evoked potential monitoring confirmed the accuracy of the preoperative fMRI in both cases (100%). The mean duration of our fMRI studies was 81.4 min (range: 45–110 min). No complications or adverse effects were observed during the performed fMRI studies.

In regard to the intraoperative DCS, identification of motor and sensory cortical areas was possible in all patients. The identification of the central sulcus was possible in 79/87 cases (90.8%) based solely on the recorded SSEPs. The same motor and sensory tasks employed during the obtained fMRI were also utilized during DCS. Similarly, the Broca's and Wernicke's areas were successfully identified in all awake craniotomies, as well as in all extraoperative stimulation and mapping procedures. It has to be mentioned that seizures were elicited during stimulation in four cases (12.9%) during an awake craniotomy, and in another two cases (11.8%) during extraoperative stimulation and mapping studies. All cases were successfully managed with no further consequences for the patient.

The concordance rate of fMRI and intraoperative DCS studies was 91.9% (80/87 cases) for localizing the sensory-motor cortical areas, while in the remaining 8.1% the intraoperative electrophysiologic and the fMRI findings were nonconcordant. Statistical analysis of our data revealed that patients with higher preoperative and postoperative KPS scores demonstrated higher percentages of fMRI and DCS concordance (*F* value = 32.430, *P* value ≤ 0.0001, and *F* value = 22.031, *P* value ≤ 0.0001, resp.). Similarly, patients with high preoperative and postoperative Spitzer index had increased incidence of fMRI and DCS finding concordance in a statistically significant way (*F* value = 37.893, *P* value ≤ 0.0001, *F* value = 26.708, *P* value ≤ 0.0001, resp.). Furthermore, a statistical trend was established from analyzing our data regarding the tumor's histological type and the concordance of fMRI and DCS findings. It seems that patients harboring GBMs tend to have more frequently nonconcordant fMRI and DCS findings (*χ* ^2^ = 6.983, *P* value = 0.07).

 Language-associated cortical areas were accurately outlined by fMRI in 85.4% (41/48 cases), while in the remaining 14.6% (7/48 cases) the intraoperative electrophysiologic and fMRI findings were non-concordant. Interestingly, in 5 of these nonconcordant cases, the fMRI localization of the central sulcus and the motor and sensory cortical areas was accurate (Table 1). Statistical analysis of our data showed that patients with higher preoperative and postoperative KPS scores demonstrated higher percentages of fMRI and DCS concordance (*F* value = 20.797, *P* value ≤ 0.0001, and *F* value = 6.144, *P*value = 0.0169, resp.). Similarly, patients with high preoperative and postoperative Spitzer index had increased incidence of fMRI and DCS finding concordance in a statistically significant way (*F* value = 22.587, *P* value ≤ 0.0001, *F* value = 26.831, *P* value ≤ 0.0001, resp.). However, tumor histological type and grade played no statistically significant role in the observed frequency of fMRI and DCS concordance (*χ* ^2^ = 1.245, *P* value = 0.5366).

The observed mean extent of tumor resection in our current series was 96.7% (range: 80–100%). Analysis of our data demonstrated no association between the extent of resection and the histological type and grade (*F* value = 0.234, *P* value = 0.8723). Interestingly, no relationship could be established in our series between the extent of tumor resection and the occurrence of any postoperative neurological deficits (*F* value = 1.493, *P* value = 0.1011). Furthermore, new postoperative neurological deficits and/or worsening of a preoperative deficit occurred in 26/87 (29.9%) of our cases. It has to be mentioned, however, that in 14/26 of these patients their postoperative deficits were temporary and their deficits had completely resolved within the first three postoperative months, reducing our actual procedure-associated neurological morbidity to 13.8% (12/87 patients). Moreover, it has to be emphasized that further neurological improvement was observed among these 12 patients with persistent deficits within the next three months.

The immunohistochemical examination of the resected tumors showed that glioblastoma multiforme occurred in 48 cases (55.2%), anaplastic astrocytomas in 26 cases (29.9%), astrocytomas grade II in 12 patients (13.8%), and oligodendroglioma grade II in one patient (1.1%).

## 4. Discussion

Surgical extirpation is theoretically considered the ideal treatment for intracranial gliomas. However, total resection is impossible in the vast majority of cases, mainly due to the infiltrative nature of gliomas, and also due to the presence of eloquent cortex and important neuronal connections in the proximity of intracranial gliomas. It has been clearly demonstrated that intracranial glioma surgical resection reduces their mechanical mass effect on the surrounding brain, diminishes the tumor-associated edema, ensures the establishment of an accurate histological diagnosis, and induces residual tumor cells into active mitotic process, thus making them more vulnerable to any adjuvant postoperative therapy [44, 51, 60–62]. Numerous clinical researchers have shown that extensive glioma resection has been associated with prolonged survival, and better quality of life [44, 51, 60–62].

Accurate knowledge of the anatomical relationship of a glioma with its neighboring eloquent cortical areas is of paramount importance for maximizing tumor resection, minimizing the chance of postoperative neurological deficit, and thus maximizing the patient's safety. Functional MRI has been employed for more than a decade in the preoperative evaluation of patients with intracranial gliomas for identifying, and accurately localizing functional cortical networks [6, 27–31, 33, 35–55, 58, 59]. Numerous clinical series have been reported comparing fMRI with intraoperative electrophysiological stimulation studies, with varying study populations, varying tumor histological types, significantly varying fMRI protocols, and thus significantly varying accuracy results, and occasionally contradictory conclusions [6, 27–31, 33, 35–55, 58, 59]. Furthermore, a large number of the previously reported series are retrospective studies, carrying all the biases and the weaknesses of retrospective studies. Therefore, the necessity for large-scale, prospective clinical studies comparing the accuracy of fMRI to intraoperative electrophysiological mapping is more than apparent.

Functional MRI accuracy regarding localization of sensory-motor cortical areas was 91.9% in our cohort. This finding is generally comparable to the existent accuracy rates described recently in the pertinent literature [6, 40, 41, 43, 52, 59]. In one of the earliest prospective studies comparing preoperative fMRI (performed at 1.5 T MRI unit) and DCS for identifying cortical motor areas, Yousry et al. [59] reported a series of six patients with gliomas. They found that the fMRI accuracy in localizing the motor cortex was 100%, when error margin was confined to 10 mm [59]. Likewise, Mueller et al. [43] reported 100% fMRI (1.5 T MR unit) accuracy in localizing sensory-motor cortex in their study. At approximately the same time, Schulder et al. [52] reported 100% accuracy rates for fMRI (performed at 1.5 T unit) in regard to sensory-motor cortex identification.Lehéricy et al.[40] reported 92% fMRI (1.5 T MR unit) accuracy in localizing sensory-motor cortex. Li et al. [41] found 100% fMRI accuracy (performed at 3 T unit) in outlining the sensory-motor cortex. Recently, Spena et al. [6] reported 92.3% fMRI (1.5 T unit) accuracy in localizing sensory-motor cortex in their study.

Contrariwise, Atlas et al. [27] reportedthatwere unable to localize by fMRI (performed at 1.5 T unit) the sensory-motor cortex in 28.5% of their cases. They postulated that glioblastomas and high-grade gliomas may alter the obtained BOLD signal and make the outlining of the sensory-motor cortex inaccurate or even impossible [27]. Fandino et al. [30] reported only 82% fMRI (1.5 T unit) accuracy in outlining the sensory-motor cortex. It has to be emphasized, however, that both these clinical series had limited-number study populations.

We found that fMRI was accurate in 85.4% of our cases in regard to the identification and outlining of the language-associated cortical areas. These findings are generally in agreement with the previously published experience [29, 31, 33, 42, 43, 46, 49, 55, 58]. Giussani et al. [33] in a systematic review of the pertinent literature found that fMRI studies could accurately outline language-associated cortical areas in a range of 59–100%. These significantly variable accuracy rates may well be explained by the great variation of the employed fMRI and DCS language paradigms. Additionally, the patient's underlying pathology, the repetition and the confirmation of the language paradigms, as well as the educational level and the cooperation of a patient, may influence both the fMRI and DCS studies. Thus, Mueller et al. [43] reported 100% fMRI (1.5 T unit) accuracy in language-associated cortex outlining. Similarly, Yetkin et al. [58] reported 100% fMRI (1.5 T unit) accuracy in localizing language-related cortical areas in their series. Likewise, Ruge et al. [49] reported 100% concordance between fMRI (1.5 T unit) and intraoperative DCS findings in defining language associated cortical areas.FitzGerald et al.[31] reported 81% sensitivity and 53% specificity for fMRI (performed at 1.5 T unit) when the margin error was 10 mm, while the respective percentages were 92% and 0% when the margin error was 20 mm. Pouratian et al. [46] reported their experience from employing preoperative fMRI (performed at 3 T unit) for localizing language-associated cortical areas. Their fMRI sensitivity and specificity rates were 100% and 66.7%, respectively, for the frontal lobe, while the respective percentages for the temporal and parietal lobes were 96.2% and 69.8% [46]. They reported 59% sensitivity and 97% specificity for fMRI, and they concluded that fMRI cannot be used alone for surgical planning, in critically-located tumors in language-associated cortical areas [46]. Similarly, Bizzi et al. [29] reported 80% sensitivity and 78% specificity for language fMRI (performed at 1.5T unit) in their series. Contrariwise, Lurito et al. [42] reported good but imperfect correlation between fMRI (performed at 1.5 T unit) and DCS findings regarding language-associated cortical areas. Similarly, Tomczak et al. [55] reported their experience from employing preoperative fMRI (performed at 1.5 T unit) and intraoperative DCS for outlining language-associated cortical areas in a large glioma series. They found only 33.3% concordance between the fMRI and DCS findings in their series [55].

Interestingly, analysis of the statistical data of our series showed that in patients with higher preoperative KPS scores and Spitzer indices, fMRI accuracy was higher in a statistically significant way. This may well be explained by the fact that patients with higher KPS and Spitzer scores were more cooperative during fMRI studies and were able to more efficiently execute the utilized sensory, motor, and language fMRI tasks. There was also a statistical trend in observing higher fMRI accuracy in patients with less malignant tumors. This finding may be related to the increased neovascularization of higher grade tumors and the potential alteration of the obtained BOLD signal. This is an important point that needs to be taken into consideration, in order to minimize the chance of inaccuracies and erroneous interpretations of the obtained preoperative fMRI studies.

The concordance rate between fMRI and DCS in regard to the localization of the visual cortex was excellent (100%) in our series, although the number of occipital cases was very limited (only two patients). Similarly, Hirsch et al. [36] reported their results from a large prospective study of 125 patients with various intracranial pathologies (including, in a large proportion, gliomas), and of 63 healthy volunteers. All their participants underwent fMRI at 1.5 T unit for sensory-motor, language, and visual cortex localization [36]. They were able to localize in the obtained fMRI the central sulcus in 100% of their healthy volunteers and in 98.4% of their patients [36]. They were also able to localize by fMRI the Wernicke's and Broca's areas in 91% and 77%, respectively, in their patients, while the respective percentages were 100% and 93% for their healthy volunteers [36]. The primary visual cortex was identified by fMRI in 100% of their tested cases [36]. It has to be emphasized, however, that further studying is mandatory before extracting any meaningful conclusions regarding fMRI's accuracy in outlining the visual cortical areas.

The observed mean resection rate in our cohort was 96.7%, while there was no statistically significant association between the extent of resection and the tumor's histological grade. Moreover, no statistically important association was established in our current series between the extent of resection and the occurrence of any new postoperative deficits. It could be postulated that performing fMRI and DCS resulted in more aggressive tumor resection without compromising however the patient's neurological status. Characteristically, worsening of the postoperative KPS scores occurred in only 2.3% of our patients, while the respective percentage of postoperative Spitzer indices was even lower (1.1%). Krishnan et al. [39] reported their results from a prospective study including 54 patients with various intracranial tumors. All their patients underwent preoperative fMRI at 1.5T and intraoperative DCS and SSEP monitoring for localizing sensory-motor cortical areas [39]. They evaluated their resection rate as well as their morbidity incidence in association with the distance of the resection margin from the fMRI defined motor cortex [39]. They found that when this distance was > 15 mm their resection rate was 85.7%, while 53% of their patients remained neurologically unchanged and 47% were improved postoperatively [39]. When this distance was between 10–15 mm, total tumor resection was achieved in 86.6% of their patients, and 13.3% developed new postoperative neurological deficits, 60% remained unchanged, while 37.3% were improved [39]. When the distance was between 5–10 mm, total resection was observed in 83.3%, while 50% of their patients remained neurologically stable, and the remaining 50% were improved postoperatively [39]. Finally, when this distance was between 0–5 mm, total resection was accomplished in 85%, neurological worsening occurred in 35% of their patients, 50% remained stable, while 15% were improved [39]. Contrariwise, Berntsen et al. [28] reported significantly lower tumor resection rates in their series. They reported that in 42% of their cases more than 95% of the tumor was resected, while their mean residual tumor was 11% (range: 0–94%) [28]. They also reported that 88% of their patients had stable postoperative neurological status, while 12% experienced some worsening [28].The-tumor-to-the-adjacent-eloquent-area distancewas related to the amount of tumor residual, and this relationship was statistically significant in their series [28].

Functional MRI provides the opportunity for noninvasively mapping cerebral cortex and outlining functional networks, thus allowing aggressive but safe tumor resection even in cases of close proximity to highly-eloquent areas. However, it carries several weaknesses and has numerous technical limitations. A totally cooperative patient is an absolute requirement since any motion artifacts (head movement, cardiac pulsation, and respiratory movements) may greatly influence its accuracy. Moreover, the educational level of the patient may interfere with the fMRI's quality, since many of the tasks, especially those related to language evaluation, require the patient's ability to comprehend and successfully execute them. It also has to be emphasized that fMRI is a time-consuming examination. Additionally, the quality of the obtained fMRI and its accuracy depend on the designing, the characteristics, and the efficacy of the utilized task paradigms every time, and this parameter needs to be taken into consideration during the fMRI interpretation [28].

Furthermore, special glioma-associated conditions may predispose to fMRI inaccuracies. It has been postulated that the presence of pathological neovascularization, particularly in cases of high-grade gliomas, may alter the obtained BOLD signal, and thus may cause false positive activations and fMRI inaccuracies [27, 33, 39, 53, 63]. It also has been described the induction of cytosol neurochemical changes in high grade gliomas with increased concentrations of nitric oxide as well as altered concentrations of adenosine 59-triphosphate, lactate, and glucose, which may influence the obtained BOLD signal and consequently distort the obtained fMRI study [27, 33, 35, 39]. In addition, the presence of tumor-associated arteriovenous shunting, and the presence of tumor-induced edema and mass effect causing mechanical vasoconstriction, as well as the presence of scar tissue secondary to a previous craniotomy, may result into significant BOLD signal changes [27, 33, 35, 39]. It has been previously demonstrated that in 10–31% of the performed fMRI studies the obtained data cannot be processed, while this percentage in solely glioma series ranges between 0–30% [35]. Moreover, gliomas are usually surrounded by edema and cause mass effect. During the fMRI study, the activation area is located in the brain tissue that may be displaced by the lesion. At the time of the craniotomy, decompression of the brain occurs and alteration of the measured distances between the activation area and the brain tumor may take place. Furthermore, it is well known that fMRI is based on magnetic susceptibility, and thus the presence of a hemorrhage within a brain tumor may alter the accuracy of the BOLD effect and misplace the location of the detected signal on the fMRI. All these parameters have to be taken into consideration for avoiding any fMRI inaccuracies and their potential clinical implications.

 The wider clinical application of higher magnetic fields may further increase the quality and consequently the accuracy of the preoperative fMRI. It has been demonstrated that higher magnetic fields may increase the obtained BOLD signal and thus may further improve fMRI's quality and accuracy [33, 37, 38, 41, 54]. Additionally, higher strength magnetic fields allow fMRI studies to be performed in shorter times and provide the opportunity for almost real time imaging of cortical activation during stimulation [64]. Moreover, the development of more concrete paradigms and protocols may further improve fMRI's accuracy and reproducibility. It has been previously demonstrated that loud language tasks provide more accurate fMRI data than silent tasks [45]. Therefore, the development of multistage, loud language tasks examining several aspects of language may further increase fMRI's accuracy. The complementary implementation of other advanced MR imaging techniques such as Diffusion Tensor Imaging (DTI) and intraoperative fMRI and DTI allow better identification and localization not only of the cortical language-associated centers but also their interconnecting networks, and thus make glioma resection safer [65]. Additionally, the wide application of intraoperative MRI and the continuously increasing usage of 5-Amino Levulinic Acid (ALA) and other identifying tumor borders techniques have increased the extent of glioma resection rates, while they have minimized the possibility of any postoperative neurological deficits [66–70]. Furthermore, the employment of resting-state fMRI may resolve the BOLD effect alteration caused in gliomas by microvascular and neurochemical cellular pathological changes [71]. These improvements in association with the designing and the development of paradigms and protocols for testing higher cognitive functions such as memory, emotion, executive thinking, and other high-cognitive functions may further increase the applicability of fMRI in the preoperative evaluation of patients with intracranial gliomas.

## 5. Conclusions

Preoperative fMRI successfully identified and outlined motor, sensory, visual, and language-associated cortical networks in all cases in our prospective series. The accuracy of the fMRI was compared to intraoperative electrophysiological mapping via DCS along with evoked potential monitoring. Functional MRI was extremely accurate in localizing motor (91.9%), sensory (91.9%), and visual (100%) cortical areas, while its accuracy in outlining language-associated cortical areas was 85.4%. Functional MRI was more accurate in patients with better neurological and functional preoperative status, while it seemed that patients with higher grade gliomas tend to have fMRI inaccuracies more frequently than patients with low grade gliomas. The employment of fMRI along with intraoperative DCS allowed us to achieve an extremely high resection rate (mean: 96.7%). Worsening of any preoperative neurological deficits and/or occurrence of a new postoperative deficit was observed in 13.8% of our cases at the completion of the third postoperative month, while worsening of the preoperative functional status occurred in only 2.3% in our series. The accuracy rate of fMRI may be further increased by employing solid paradigms, preferably the same paradigms for fMRI and DCS whenever possible, and by ruling out any conditions which could cause fMRI inaccuracies. Although fMRI cannot replace DCS for localizing cortical eloquent areas, when employed in association with DCS can guide and increase DCS's efficacy and provide valuable information for better surgical planning and safer resection of intracranial gliomas.

## Figures and Tables

**Table 1:** Summary of the demographic data, pre and postoperative neurological and functional scores, fMRI and electrophysiological employed tests, concordance of their findings, extent of tumor resection, and tumor histological type and grade (C: concordant, NC: nonconcordant, AA: anaplastic astrocytoma, GBM: glioblastoma multiforme).

Patient	Age	Gender	Tumor location	fMRI tests	fMRI duration in min	DCS tests	Other neurophysiological tests	Preop. KPS	Postop. KPS	Preop. Spitzer index	Postop. Spitzer index	Postop. neurological deficit	Concordance of fMRI and DCS regarding motor/sensory cortex	Concordance of fMRI and DCS regarding language	Tumor histology	Extend of resection %
JK	65	F	L temporal	Language, motor	94	Motor, sensory, language	MEP, SSEP	100	100	10	10	None	C	C	GBM	100
GH	58	M	L temporal	Language, motor	87	Motor, sensory, language	MEP, SSEP	100	100	10	10	None	C	C	GBM	95
RG	67	M	R parietal	Motor, sensory	62	Motor, sensory	MEP, SSEP	90	100	10	10	None	C	N/A	AA	95
DF	49	F	L temporal	Language, motor	101	Motor, sensory, language	MEP, SSEP	80	90	7	8	Improving conductive aphasia	NC	NC	GBM	100
VJ	72	F	L temporal	Language, motor, sensory	98	Motor, sensory, language	MEP, SSEP	100	100	10	10	None	C	C	GBM	100
DR	59	M	R parietal	Motor, sensory	70	Motor, sensory	MEP, SSEP	80	100	8	8	None	C	N/A	Astrocytoma II	90
TH	60	F	L frontal	Language, motor, sensory	100	Motor, sensory, language	MEP, SSEP	90	90	10	10	Improving expressive aphasia	C	C	GBM	100
KM	67	F	L frontal	Language, motor, sensory	105	Motor, sensory, language	MEP, SSEP	100	100	10	10	None	C	C	AA	100
AT	58	M	L temporal	Language, motor, sensory	99	Motor, sensory, language	MEP, SSEP	80	90	8	8	Improving conductive aphasia	C	NC	AA	95
FN	64	F	L temporal	Language, motor, sensory	100	Motor, sensory, language	MEP, SSEP	90	100	9	9	None	C	C	GBM	100
IR	70	F	L temporal	Language, motor, sensory	108	Motor, sensory, language	MEP, SSEP	100	100	10	10	None	C	C	GBM	95
WR	50	F	L frontal	Language, motor, sensory	105	Motor, sensory, language	MEP, SSEP	90	100	10	10	None	C	C	AA	100
RR	69	F	L parietal	Motor, sensory	62	Motor, sensory	MEP, SSEP	70	90	6	8	Improving R hemiparesis	NC	N/A		95
EB	70	F	L frontal	Language, motor, sensory	104	Motor, sensory, language	MEP, SSEP	80	90	7	7	Improving expressive aphasia	C	NC	GBM	100
KL	74	F	L temporal	Language, motor, sensory	100	Motor, sensory, language	MEP, SSEP	90	90	8	9	Balance difficulties, gait problems	C	C	GBM	100
TW	70	M	R parietal	Motor, sensory	70	Motor, sensory	MEP, SSEP	80	90	9	9	Improving L monoparesis	C	N/A	GBM	90
DF	67	F	R parietal	Motor, sensory	61	Motor, sensory	MEP, SSEP	90	100	9	9	None	C	N/A	GBM	100
LP	68	M	L frontal	Language, motor, sensory	96	Motor, sensory, language	MEP, SSEP	70	80	6	6	Personality disorders	C	C	AA	100
TB	61	F	L parietal	Motor, sensory	67	Motor, sensory	MEP, SSEP	90	100	8	8	None	NC	N/A	GBM	85
KD	49	M	L parietal	Motor, sensory	62	Motor, sensory	MEP, SSEP	100	100	10	10	None	C	N/A	AA	95
SF	39	F	L temporal	Language, motor, sensory	98	Motor, sensory, language	MEP, SSEP	100	100	10	10	None	C	C	Astrocytoma II	100
EH	68	M	L frontal	Language, motor, sensory	95	Motor, sensory, language	MEP, SSEP	90	100	10	10	None	C	C	GBM	100
TP	59	M	L parietal	Motor, sensory	58	Motor, sensory	MEP, SSEP	80	80	8	8	R hemiparesis	C	N/A	Astrocytoma II	90
SR	70	F	R parietal	Motor, sensory	61	Motor, sensory	MEP, SSEP	70	80	6	7	Improving L monoparesis	NC	N/A	GBM	100
LR	60	F	L parietal	Motor, sensory	67	Motor, sensory	MEP, SSEP	90	100	8	8	None	C	N/A	GBM	100
HG	59	M	R temporal	Language, motor, sensory	102	Motor, sensory, language	MEP, SSEP	100	100	10	10	None	C	C	AA	100
FR	65	F	L occipital	Sensory, motor, visual	59	Sensory, motor, visual	MEP, SSEP, VEP	100	100	10	10	Stable hemianopsia	C	N/A	AA	100
PR	72	F	R parietal	Motor, sensory	63	Motor, sensory	MEP, SSEP	70	80	7	7	Improving L hemiparesis	C	N/A	GBM	90
SS	68	M	R parietal	Motor, sensory	60	Motor, sensory	MEP, SSEP	90	90	8	9	L hemiparesis	C	N/A	AA	100
FM	67	M	L temporal	Language, motor, sensory	105	Motor, sensory, language	MEP, SSEP	100	100	10	10	None	C	C	AA	90
AO	40	M	L parietal	Motor, sensory	65	Motor, sensory	MEP, SSEP	100	100	10	10	None	C	N/A	Astrocytoma II	90
DN	57	F	L temporal	Language, motor, sensory	104	Motor, sensory, language	MEP, SSEP	100	100	10	10	None	C	C	AA	100
MM	56	M	L parietal	Motor, sensory	60	Motor, sensory	MEP, SSEP	100	100	10	10	None	C	N/A	Astrocytoma II	95
KR	69	M	R parietal	Motor, sensory	58	Motor, sensory	MEP, SSEP	90	100	8	9	None	C	N/A	GBM	100
RS	74	M	R parietal	Motor, sensory	55	Motor, sensory	MEP, SSEP	70	80	7	7	L hemiparesis	NC	N/A	GBM	95
MA	59	M	L temporal	Language, motor, sensory	95	Motor, sensory, language	MEP, SSEP	100	100	10	10	None	C	C	Astrocytoma II	100
NN	76	F	L frontal	Language, motor, sensory	110	Motor, sensory, language	MEP, SSEP	80	90	8	8	Improving expressive aphasia	NC	NC	GBM	95
SG	69	M	R parietal	Motor, sensory	62	Motor, sensory	MEP, SSEP	90	90	10	10	L monoparesis	C	N/A	GBM	95
CT	56	M	L temporal	Language, motor, sensory	105	Motor, sensory, language	MEP, SSEP	100	100	10	10	None	C	C	AA	95
ZT	34	M	R parietal	Motor, sensory	60	Motor, sensory	MEP, SSEP	100	100	10	10	None	C	N/A	Astrocytoma II	100
GK	66	M	R parietal	Motor, sensory	64	Motor, sensory	MEP, SSEP	90	100	9	9	None	C	N/A	AA	95
TW	60	F	L frontal	Language, motor, sensory	100	Motor, sensory, language	MEP, SSEP	80	80	8	9	Personality disorders	C	C	GBM	100
HT	47	M	L temporal	Language, motor, sensory	104	Motor, sensory, language	MEP, SSEP	100	100	10	10	None	C	C	Astrocytoma II	100
EJ	58	M	L temporal	Language, motor, sensory	99	Motor, sensory, language	MEP, SSEP	100	100	10	10	None	C	C	GBM	95
PR	53	M	L frontal	Language, motor, sensory	103	Motor, sensory, language	MEP, SSEP	100	100	10	10	Mild abulia	C	C	AA	100
PS	67	F	L parietal	Motor, sensory	65	Motor, sensory	MEP, SSEP	90	90	8	8	R monoparesis	C	N/A	GBM	95
RJ	62	F	L frontal	Language, motor, sensory	95	Motor, sensory, language	MEP, SSEP	100	100	10	10	None	C	C	GBM	100
CV	50	F	R temporal	Language, motor, sensory	98	Motor, sensory, language	MEP, SSEP	100	100	10	10	None	C	C	AA	100
VP	33	F	L parietal	Motor, sensory	65	Motor, sensory	MEP, SSEP	90	100	8	9	None	C	N/A	Oligodendroglioma II	100
RC	62	M	L frontal	Language, motor, sensory	100	Motor, sensory, language	MEP, SSEP	100	100	10	10	None	C	C	GBM	100
JK	74	F	R parietal	Motor, sensory	58	Motor, sensory	MEP, SSEP	80	70	7	8	Worsening of L hemiparesis	NC	N/A	GBM	90
AT	56	M	L frontal	Language, motor, sensory	102	Motor, sensory, language	MEP, SSEP	100	100	10	10	None	C	C	Astrocytoma II	100
WR	60	M	L temporal	Language, motor, sensory	100	Motor, sensory, language	MEP, SSEP	100	100	10	10	Temporary mild dysphasia	C	C	Astrocytoma II	100
PH	50	M	L temporal	Language, motor, sensory	98	Motor, sensory, language	MEP, SSEP	100	100	10	10	None	C	C	AA	95
DK	67	M	L parietal	Motor, sensory	59	Motor, sensory	MEP, SSEP	100	100	10	10	None	C	N/A	AA	95
LP	56	M	L frontal	Language, motor, sensory	102	Motor, sensory, language	MEP, SSEP	100	100	10	10	Premotor syndrome	C	C	AA	95
DN	76	M	L temporal	Language, motor, sensory	100	Motor, sensory, language	MEP, SSEP	90	90	8	9	Gait difficulties	C	C	GBM	100
AS	70	M	L parietal	Motor, sensory	58	Motor, sensory	MEP, SSEP	100	100	10	10	None	C	N/A	GBM	100
VS	55	F	L temporal	Language, motor, sensory	95	Motor, sensory, language	MEP, SSEP	100	100	10	10	None	C	C	AA	100
DK	55	M	L parietal	Motor, sensory	60	Motor, sensory	MEP, SSEP	100	100	10	10	None	C	N/A	AA	95
AK	70	M	L frontal	Language, motor, sensory	104	Motor, sensory, language	MEP, SSEP	90	90	8	8	Abulia, urinary incontinence	C	NC	GBM	100
KV	69	M	L frontal	Language, motor, sensory	95	Motor, sensory, language	MEP, SSEP	100	100	10	10	Personality disorders	C	C	GBM	100
KP	73	F	L temporal	Language, motor, sensory	100	Motor, sensory, language	MEP, SSEP	90	90	8	8	Stable Dysphasia	C	NC	GBM	100
KM	72	M	L frontal	Language, motor, sensory	93	Motor, sensory, language	MEP, SSEP	90	90	9	8	Stable personality disorders	C	C	GBM	95
VP	66	M	R parietal	Motor, sensory	55	Motor, sensory	MEP, SSEP	90	100	8	8	Improving L monoparesis	C	N/A	GBM	95
EZ	70	M	L frontal	Language, motor, sensory	95	Motor, sensory, language	MEP, SSEP	100	100	10	10	Temporary dysphasia	C	C	GBM	95
VT	70	M	L temporal	Language, motor, sensory	100	Motor, sensory, language	MEP, SSEP	100	100	10	10	None	C	C	GBM	100
PV	67	F	R parietal	Motor, sensory	53	Motor, sensory	MEP, SSEP	80	80	9	9	Stable L hemiparesis	C	N/A	GBM	85
KK	69	M	L temporal	Language, motor, sensory	95	Motor, sensory, language	MEP, SSEP	100	100	10	10	None	C	C	GBM	100
MC	56	M	L parietal	Motor, sensory	55	Motor, sensory	MEP, SSEP	100	100	10	10	Temporary R monoparesis	C	N/A	AA	100
SC	64	M	R parietal	Motor, sensory	57	Motor, sensory	MEP, SSEP	100	100	10	10	None	C	N/A	GBM	95
EV	48	M	L temporal	Language, motor, sensory	100	Motor, sensory, language	MEP, SSEP	100	100	10	10	None	C	C	AA	95
KG	70	M	R parietal	Motor, sensory	50	Motor, sensory	MEP, SSEP	90	90	8	8	Stable L hemiparesis	C	N/A	GBM	100
MK	69	M	L parietal	Motor, sensory	58	Motor, sensory	MEP, SSEP	100	100	10	10	None	C	N/A	Astrocytoma II	100
CT	75	F	L frontal	Language, motor, sensory	100	Motor, sensory, language	MEP, SSEP	80	70	7	7	Confusion, personality disorders, flat affect	NC	C	GBM	100
JH	48	M	R parietal	Motor, sensory	50	Motor, sensory	MEP, SSEP	90	100	8	8	None	C	N/A	AA	95
PG	55	M	R temporal	Language, motor, sensory	106	Motor, sensory, language	MEP, SSEP	100	100	10	10	None	C	C	GBM	100
ES	58	M	L parietal	Motor, sensory	55	Motor, sensory	MEP, SSEP	100	100	10	10	None	C	N/A	AA	90
EA	72	F	R parietal	Motor, sensory	45	Motor, sensory	MEP, SSEP	70	80	6	7	Improving L hemiparesis	C	N/A	GBM	80
VT	60	M	L parietal	Motor, sensory	50	Motor, sensory	MEP, SSEP	90	100	8	8	Improving R monoparesis	C	N/A	GBM	100
SK	55	M	L frontal	Language, motor, sensory	100	Motor, sensory, language	MEP, SSEP	80	90	8	8	Improving personality disorder	C	NC	AA	100
JF	65	F	L frontal	Language, motor, sensory	105	Motor, sensory, language	MEP, SSEP	100	100	10	10	None	C	C	GBM	95
GL	66	F	L occipital	Sensory, motor, visual	50	Sensory, motor, visual	MEP, SSEP,VEP	100	100	10	10	Stable visual hemianopsia	C	N/A	GBM	100
TS	70	M	R parietal	Motor, sensory	55	Motor, sensory	MEP, SSEP	80	80	8	8	Stable L hemiparesis	C	N/A	GBM	85
LF	59	M	L temporal	Language, motor, sensory	95	Motor, sensory, language	MEP, SSEP	100	100	10	10	None	C	C	AA	95
MI	70	F	L parietal	Motor, sensory	55	Motor, sensory	MEP, SSEP	70	90	6	7	Improving R hemiparesis	NC	N/A	GBM	100
MA	75	F	L temporal	Language, motor, sensory	90	Motor, sensory, language	MEP, SSEP	80	80	7	8	Gait difficulties	C	C	GBM	85
